# Interaction and Emotional Connection with Pets: A Descriptive Analysis from Puerto Rico

**DOI:** 10.3390/ani10112136

**Published:** 2020-11-17

**Authors:** Ursula Aragunde-Kohl, José Gómez-Galán, Cristina Lázaro-Pérez, José Ángel Martínez-López

**Affiliations:** 1 Department of Psychology, Ana G. Méndez University, Gurabo Campus, Gurabo, PR 00777, USA; aragundeu1@uagm.edu; 2 Department of Education, University of Extremadura, Avda, de Elvas, s/n, 06006 Badajoz, Spain; 3 College of Education, Ana G. Méndez University, Cupey Campus, San Juan, PR 00926, USA; 4 Department of Sociology, University of Murcia, C/Campus Universitario, 11, 30100 Murcia, Spain; cristina.lazaro2@um.es; 5 Department of Social Work and Social Services, University of Murcia, Avda, Teniente Flomesta, 5, 30003 Murcia, Spain; jaml@um.es

**Keywords:** human–animal interaction, companion pets, psychological health, welfare, animal-assisted intervention, animal protection, education, human–animal bond

## Abstract

**Simple Summary:**

The coexistence of humans with pets, and the type and quality of interactions that derive from it, is a growing line of research that covers many scientific disciplines: veterinary sciences, education, psychology, biological sciences, sociology, neuroscience, etc. In this article, we analyze both the characteristics of care for pets and the feelings and emotions involved in the human–pet relationship in the population of Puerto Rico. The sample has been of 1436 people. The most representative results of the research show how pets have a positive impact on the mental health of their owners, because of the emotional connection established, which leads them to experience mainly feelings of love, joy and peace.

**Abstract:**

The study of human–animal interactions has become a prominent research field. The presence of pets in our daily lives has meant a change in the perception of our relationship with pets. One of the new lines of research that has opened up in this area analyzes the possible physical, emotional, and psychological benefits of a human–animal bond. In this context, two main objectives were pursued in this study, whose sample (*N* = 1436) was investigated in Puerto Rico: (PO1) determining the characteristics of the owners and the care of the pets within Puerto Rican society and (PO2) to analyzing the benefits of living with pets for health and personal well-being, especially at the psychological level. We sought to identify the feelings that arose in pet caretakers as a result of human–animal interactions. The methodology used, which was descriptive and not experimental, was based on a questionnaire of 86 questions with a Likert scale. After the descriptive analysis was undertaken through a frequency analysis, a binary logistic regression was carried out to establish whether the feelings and emotions of the subjects toward their companion pets were determined by sociodemographic variables and were related to the type of coexistence with their pets. The two main results from this study are as follows: for most owners, their pets are very important beings, and those who establish an emotional connection with their pets experience love, joy, and peace. Pets are part of human life through very intense relationships and interactions that, in most cases, increase personal welfare by providing improvements to the human–animal biopsychosocial system. In this sense, a relationship with a pet has a positive impact on the mental health of the owner. Pets are a part of our family lives and are regulators of the most elementary feelings and emotions in humans.

## 1. Introduction

Research on the human–animal bond has been recognized as a legitimate part of studying human interactions over the past few decades [1,2,3,4,5]. The relationships we form with animals and how we interact with each species is influenced by multiple factors, such as individual preferences and social and cultural norms [6,7,8]. This has been especially intensified because of our personal experiences with pets. Research has allowed us to better understand this connection in our everyday lives, where several authors have highlighted that to understand this human–animal bond, we need to better understand these factors and how they impact the relationships between humans and non-human animals in different contexts around the globe [9,10,11,12].

The concept known as human–animal interaction (H–AI) has been established in the literature, where the possible benefits of animal companions have been investigated in terms of the physical, emotional, and psychological aspects of well-being [13,14]. According to Palley, O’Rourke, and Niemi [15], this interaction generally has a positive impact on the lives of humans, enhancing our well-being and bolstering our resilience in difficult life circumstances. Specifically, some of the identified benefits are as follows: (a) An increase in good humor, laughter, and play; (b) an increased body relaxation state; (c) body contact and unconditional emotional closeness; (d) an increase in social interactions; (e) the provision of a conversation partner and an important family member [16]. As such, this research is a new scientific dimension with a multidisciplinary nature that is making its way into the scientific, academic, and educational fields [17,18].

Furthermore, research has also suggested that interactions with pets over the long term can reduce the responses of the autonomous nervous system [19], increasing states of relaxation and well-being. Some possible variables that have been pointed out as mediators of this relationship include the perception of unconditional acceptance, the lack of judgment, and the continuous provision of love [20]. This, in turn, provides a sense of security and promotes emotional openness in ourselves [21]. Additionally, in the general population, our interactions with pets have been shown to have the general effects of minimizing feelings of loneliness, depression, and stress, among others [16,22,23,24,25,26,27,28]. Thus, the evidence suggests that when people interact with pets, it has a generally positive effect on their overall wellbeing and this, in turn, becomes an important resource to be used.

However, despite these findings, many still do not accept and welcome animal companions as a possible tool to enhance our well-being and life. Some studies highlight this issue. For example, when comparing owners and non-owners of pets, Hirsch and Whitman [29] found no differences in the case of chronic pain, while Kidd and Martinez [30] found no differences in terms of the self-acceptance and well-being scales of the California Psychological Inventory [31]. Furthermore, some studies in the scientific literature speak of negative attitudes that arise from human–animal interaction, which occur in cases of animal abuse by action [32,33,34,35]; abuse by omission, such as abandonment [36,37]; or animal accumulation disorder [38].

In Latin America, and specifically in Puerto Rico, literature about the human–animal bond is scarce [39]. However, some of the research focused specifically on animal-assisted therapy (AAT) has shown openness from health professionals to include pets as part of healing processes [40]. However, what we have seen there are only a few professionals that include questions regarding companion pets or provide social and community spaces that allow for companion pets as part of normal social interactions. Rivera-Ortiz [41] showed how important relationships with an animal companion is in the lives of Puerto Rican heterosexual couples. Furthermore, recent research has shown that in a cultural context in which the family is one of the most important cultural values, the relationship between companion pets and other family members could be different from other contexts [39]. Thus, a better understanding of these relationships in different cultural contexts is vital.

Therefore, in light of this gap in the scientific literature, the purpose of our study is to explore the interactions, attitudes, and possible links to companion animals in Puerto Rico that can generate and improve the welfare of pet caretakers and become a protective factor [42,43,44,45].

## 2. Materials and Methods

### 2.1. Objectives

This research investigated the spectrum of coexistence with pets and the interactions that occur with humans, pursuing two main objectives: (PO1) to provide an approach for describing the profile of pet caretakers and the characteristics of the care of pets by Puerto Rican society, and (PO2) since numerous studies have been done in the past decade that highlight the implications for the health and personal welfare of people living with pets [46,47,48,49,50,51,52,53,54,55], we investigated whether there are predictive variables regarding the feelings that emerge in caretakers as a result of the interaction with their pets.

### 2.2. Instruments

The main instrument used for this research was the “Puerto Rico Comprehensive Companion Animal Survey” questionnaire, which was validated and determined as reliable according to the usual protocols used in the social sciences [56]. A first draft was sent to experts in the field of animal welfare, psychology, and linguistics for their revision and recommendations regarding possible biases, content, grammatical errors, and ease of understanding.

The questionnaire had a total of 86 questions, 60 of which were multiple-choice, and 26 were statements rated using a five-point Likert scale (from 1—strongly disagree to 5—strongly agree). The questionnaire was divided into six sections. Section I included questions regarding sociodemographic information. Section II consisted of questions regarding keeping companion animals in terms of how many types of, and reasons for obtaining companion pets; companion animal keeping practices; attachment level to their companion pets. Section III consisted of questions regarding the situation of stray dogs and cats. Section IV included statements about nonhuman animal sensitivity and intelligence perceptions. Section V included statements regarding the needs and behaviors of animal companions. Section VI included statements regarding compassion toward pets and Section VII included statements regarding violence toward pets and Puerto Rican animal protection laws.

### 2.3. Variables Used

Dependent variable: for the first objective (PO1), we used the coexistence and the characterization of pet care by the Puerto Rican population. For the second objective (PO2), the dependent variables were the feelings that the pets brought about in people.

Independent variables: the independent variables were grouped into four different categories, namely, socio-demographic, human and animal companion caretaking, the human–animal companion bond, and subjective perceptions about the feelings aroused by interactions with pets. The following sociodemographic variables were used: (a) gender, (b) marital status, (c) age, (d) highest schooling grade completed, (e) type of housing, (f) annual income, and (g) current physical health rating. Regarding pet caretaking, the following information was gathered: (a) the primary caretaker of the pet, (b) the types of food the pet gets, (c) time spent with the pet, (d) where the pet sleeps, and (e) the primary caretaker of the pet. Regarding the human companion bond, the following categories were used: (a) activities with pets, (b) the main reason for having a pet, and (c) whether the pet is part of the family. The existence/absence of these categories was established through closed-ended multi-response questions that indicated what feelings emerged in people after interacting with their pets. Therefore, while what these feelings meant were not previously quantified (through a scale or similar), in a subjective way and according to their consideration and experiences, the participants expressed their feelings regarding their pets.

### 2.4. Sample and Participants

The target population of this survey was Puerto Rican residents aged at least 21 years old. The participants were a representation of the general population in Puerto Rico, regardless of their views about companion pets.

A total of 1436 responses were collected through the survey. Females represented the majority (79%) of participants. The respondents were mostly from the metropolitan area of Puerto Rico, where almost 69% stated that they were from urban areas. The economic status data showed that most participants (66%) had an annual income below $30,000. The rest of the relevant information about the participants is shown in Table 1.

### 2.5. Procedures

The protocol followed all the guidelines of the ethics committees of the universities to which the members of the research team belonged. The study and all protocols were submitted and approved by the Turabo University Institutional Review Board (IRB; investigation number: 03-336-12). The survey was uploaded to a provider of web-based survey solutions and was also provided in a paper format. A written statement about the research in the form of an information sheet was provided to and read by each participant before completing the survey. Each survey did not start unless the participant indicated that they had read and understood the information sheet. Thus, all participants (*n* = 1436) gave their informed consent in accordance with the Declaration of Helsinki.

The study was distributed in a paper format and was disseminated with a flyer to different community centers, a diverse university campus, and relevant social networking sites, e.g., Facebook. Email distribution lists were also used, including the SUAGM (*Sistema Universitario Ana G. Méndez*, today UAGM, *Universidad Ana G. Méndez*) university global distribution lists, the Puerto Rican Psychological Association, student associations, animal welfare groups, and shelters. The general population, including dog owners and non-dog-owners, were invited to take part. All participants completed either the paper or web-based questionnaire voluntarily, where the questionnaire was made available in both formats for about six months.

The data were analyzed using IBM’s Statistical Package for the Social Sciences v.24 (SPSS ©, IBM, Armonk, New York, NY, USA). Descriptive analyses were undertaken for all variables. The procedure was structured into two phases. First, a descriptive analysis was carried out through a frequency analysis. Subsequently, a binary logistic regression was performed to establish whether the feelings generated in the subjects during their interactions with their pets were determined by sociodemographic variables and related to the coexistence with them. Through logistic regression, it was possible to know which variables were predictive and the intensities of their effects. The feelings studied were love, peace, sadness, joy, tranquility, relief, support, anxiety, protection, and stress. The existence of these feelings was established in a dichotomous way (yes/no) from the participants’ statements.

The following sociodemographic variables were established for the analysis of the binary logistic regression: (a) sex (female/male), (b) age (21–35 years, 36–55 years, and ≥56 years), (c) marital status (married, living with a partner, single, divorced, separated, living together), (d) annual income level (up to $20,000, $20,001–50,000, and ≥$50,001), and (e) maximum level of studies achieved (up to the fourth year of high school, associate’s degree or bachelor’s degree). As for the coexistence with pets, two variables were established: (a) coexistence with pets and (b) the number of pets the participant coexisted with (0/1/2/3/4/5+).

In the binary logistic regression, the forward model was used, which automatically re-evaluates the coefficients and their significance, eliminating those variables from the model that are not statistically significant [57].

## 3. Results

The data were collected from 1436 self-selected participants over six months. However, the data from 109 participants were discarded because they did not answer the questions required to complete the questionnaire; thus, a total of 1327 responses were obtained.

Table 2 shows that 84% of the participants indicated they had at least one pet in their home, while only 16% said they had none. Participants specified that they obtained their pets in different ways: 35% were a gift, 32% were rescued, 23% were bought, and 10% were adopted. Additionally, almost 82% of participants currently had a total of 4 (mean = 3.8) animal companions. The majority had a preference for dogs (46%), cats (45%), birds (45%), and fishes (42%).

Overall, 68% of the participants indicated that they and another family member were responsible for taking care of the basic needs of their companion pets; only 22% indicated that they were the sole caretaker of their companion animal. The majority (88%) of the participants indicated that their companion animals received food specific to the species. Complete information on animal caretaking is shown in Table 3.

The majority (40%) of the participants indicated that they spent 12 h or more with their companion pets each day. Regarding sleeping arrangements, 33% said their companion pets slept in their bedroom, 32% slept inside the house, and 23% slept in the yard without restraint.

Table 4 presents a summary of some of the general components of the human–animal companion bond. Concerning the activities undertaken with their companion pets, participants said that they stroked (94%), played with (92%), talked to (89%), and walked (57%) their pets. Regarding the reasons for having a pet, 91% did so for company, 42% for pleasure, and 40% for safety. They also rated their pets as family members (99%).

Finally, to determine the existence of predictive variables regarding the feelings that people experience toward their pets, we proceeded to carry out a binary logistic regression of some of the most frequent emotions that arose in the pet owners, both positive and negative, that were given in the questionnaire. Regarding the positive feelings, the following were considered: love, peace/support, joy, tranquility, relief, and protection; regarding the negative feelings, the following were considered: sadness, anxiety, and stress.

The logistic regression model was statistically significant in the case of love (χ^2^ = 85.119, *p* < 0.005). The model explained 14.2% (Nagelkerke’s R^2^) of the variance and correctly classified 90.7% of the cases. The Hosmer–Lemeshow test showed that there were no significant differences between the observed and predicted results in the model, with *p* = 0.806.

As for the variables predicting events, the following were significant: (a) lives with a pet and (b) marital status. Pet ownership had an OR = 20.152, 95% CI = 10.443–38.888, *p* = 0.000. This result showed the strong predictive value of this variable through the love that pets awaken in their owners simply by living with an animal; in fact, pet ownership increased the chances of feeling love by up to 20 times. Regarding marital status, the results were significant in the cases of living as a couple and being single. Specifically, the OR for people living with a partner was 1.969, 95% CI = 1.083–3.582, *p* = 0.026. Being single presented an OR = 1.630, 95% CI = 1.005–2.642, *p* = 0.048. Thus, we observed that people who lived with a partner and those who were single were up to 1.9 and 1.6 times more likely to feel loved because of their pet(s) than those who had a different marital status.

In relation to peace, the logistic regression model was statistically significant (χ^2^ = 57.571, *p* < 0.005). The model explained 7.3% (Nagelkerke’s R^2^) of the variance and correctly classified 63.3% of the cases. The Hosmer–Lemeshow test showed that there were no significant differences between the observed and predicted results in the model, with *p* = 0.739.

There were numerous predictive variables in the case of feeling peaceful: (a) whether one lived with a pet, (b) marital status, and (c) degree. Living with a pet presented an OR = 6.450, 95% CI = 3.039–13.691, *p* = 0.000. Although this did not have as high a value as in the case of the feeling of love, it was still a remarkable result. Thus, a person who lived with a pet was almost 6.5 times more likely to feel peaceful than a person who did not live with a pet. In the case of marital status, predictive values were observed in three subcategories: living with a partner, separated/divorced, and single. Regarding the first subcategory, cohabitation as a couple presented an OR = 1.928, 95% CI = 1.339–2.775, *p* = 0.000; for separated or divorced people, OR = 1.765, 95% CI = 1.156–2.694, *p* = 0.008. For those who were single, OR = 1.959, 95% CI = 1.428–2.688, *p* = 0.000. Therefore, regarding people who were in couples, separated/divorced, or single, they were up to approximately two times more likely to feel at peace from their interactions with a pet than those who were married or widowed. Finally, regarding feeling peace, people who had a higher degree (associate’s degree, bachelor’s degree, master’s degree, or doctorate) had an OR = 1.555, 95% CI = 1.150–2.104, *p* = 0.004. As such, it was observed that feeling peace was associated to a greater extent with people who had a higher level of education, and in this specific case, when they had an associate’s or bachelor’s degree.

Regarding the feeling of joy, the logistic regression model was statistically significant (χ^2^ = 94.943, *p* < 0.005). The model explained 18.1% (Nagelkerke’s R^2^) of the variance and correctly classified 91.3% of the cases. The Hosmer–Lemeshow test showed that there were no significant differences between the observed and predicted results in the model, with *p* = 0.729.

In this case, the predictive variables were (a) living with a pet, (b) sex, and (c) degree. The results obtained regarding having a pet were relevant and conclusive with an OR = 38.094, 95% CI = 9.482–153.05, *p* = 0.000. This was undoubtedly one of the most outstanding results of this research given the association and prediction of the variable “coexistence with a pet” with a feeling as important as happiness. A person who lived with a pet was up to 38 times more likely to feel joy in their interaction with their pet than a person who did not live with a pet. Regarding sex, being a woman increased the possibility of feeling joy, presenting an OR = 2.004, CI 95% = 1.044–3.847, *p* = 0.037. In other words, women were up to twice as likely to feel joy when in the company of a pet than men. Finally, regarding degree holders, when the degree was equal to or higher than an associate’s degree, degree level was a predictive variable, recording an OR = 2.032, 95% CI = 1.260–3.275, *p* = 0.004. Therefore, people with an associate’s degree or higher were up to twice as likely to feel joy because of their pet(s) than those with a lower educational level. Furthermore, the higher their formative level, the greater the possibility of feeling joy in their interaction with their pet(s).

The logistic regression model was statistically significant in the case of relief (χ^2^ = 42.476, *p* < 0.005). The model explained 5.6% (Nagelkerke’s R^2^) of the variance and correctly classified 71.5% of the cases. The Hosmer–Lemeshow test showed that there were no significant differences between the observed and the predicted results in the model, with *p* = 0.998.

As for the variables predicting the events, the following were significant: (a) lives with a pet and (b) annual income level. Pet ownership had an OR = 4.385, 95% CI = 1.548–12.424, *p* = 0.005. In other words, living with a pet increased the relief that could be produced in negative situations, and people who lived with a pet were 4.3 times more likely to develop this feeling when they were with their pet(s) than those who did not have a pet. In relation to the level of annual income, those who had an annual income in the range of $20,001–$50,000 presented an OR = 0.566, 95% CI = 0.412–0.779, *p* = 0.000. However, their β was −0.569, where the values were calculated relative to the value considered as the reference. As such, people who had an annual income of ≥$50,001 were more likely to feel relief than those who were in the annual income range of $20,001–$50,000.

Regarding the emotional response to anxiety, the logistic regression model was statistically significant (χ^2^ = 11.190, *p* < 0.005). The model explained 6.0% (Nagelkerke’s R^2^) of the variance and correctly classified 98.0% of the cases. The Hosmer–Lemeshow test showed that there were no significant differences between the observed and predicted results in the model, with *p* = 1.

The only predictive variable was the annual income level. People with an annual income in the range of $20,001–$50,000 had an OR = 10.964, 95% CI = 1.271–94.582, *p* = 0.029. People with annual incomes up to $20,000 had an OR = 10.898, 95% CI = 1.433–82.875, *p* = 0.021. Regarding relief and anxiety, these data showed us that the levels of anxiety were significantly reduced in the case of people with a high annual income (>$50,000) and that the possibility of suffering anxiety increased by around 10 points among those with an annual income ≤$50,000.

Regarding the feeling of protection, the logistic regression model was statistically significant (χ^2^ = 42.164, *p* < 0.005). The model explained 5.3% (Nagelkerke’s R^2^) of the variance and correctly classified 63.8% of the cases. The Hosmer–Lemeshow test showed that there were no significant differences between the observed and predicted results in the model, with *p* = 1.

The variables that were shown to be predictive were (a) living with a pet and (b) annual income level. People who had a pet had an OR = 7.604, 95% CI = 3.482–16.605, *p* = 0.000. That is to say, the feeling of protection generated by living with a pet increased by 7 points relative to people who did not live with a pet. People with an annual income of up to $20,000 had an OR = 1.462, 95% CI = 1.106–1.932, *p* = 0.008. Therefore, people with lower annual incomes ($0–$20,000) experienced a greater sense of protection through their pet(s) than people with higher annual incomes.

The models of logistic regression of the emotional reactions of tranquility, sadness, support, and stress are not presented because they did not show an adequate statistical adjustment. The statistical summary of the binary logistic regressions is showed in Table 5.

## 4. Discussion

Through this research, a descriptive and explanatory approach was undertaken regarding the characteristics of care and coexistence with pets and the feelings they arouse in their interactions with humans, which are research aspects that have recently emerged in multiple dimensions and in very different specialties [58,59,60,61,62,63,64,65].

The results of our investigation showed that most of the respondents had a pet companion and usually more than one. This finding showed that Puerto Rico is part of the worldwide trend of wanting to have and including companion pets in our everyday lives [66,67].

The results we have obtained allow us to argue that pets should be included in the welfare and health debate, as most respondents have a strong connection with their companion animal(s). Many also consider their pet(s) to be part of their family. This means that we not only integrate them into our daily lives by taking care of their basic needs, such as providing water and food, but we also interact with them on emotional and physical levels. This relationship is an opportunity for many to connect on intimate and deep levels [68,69,70,71,72,73]. We develop ways to communicate, create rituals, and provide the necessary sensitivity to interact with them appropriately such that both species are benefitted [74].

The results of this study aligned with other research on the topic that shows that human–animal interaction enhances and facilitates positive traits within us [72,75,76,77], but most importantly, they permit us to create spaces to unwind, play, and be ourselves.

In our findings regarding the time spent with companion pets, the participants spent many hours with them, and in many cases, more than with other people or family members. This reflects a connection that is very intimate and personal, which is possibly augmented by the fact that most of the respondents were the main caretakers of their pets [78].

This study provided a first glance showing that the human–animal bond in Puerto Rican communities is very strong and important [79]. It suggests that people interact and include their companion animal in everyday activities (via daily company, sleeping arrangements, and leisure activities), which has a positive effect on both participants (human and animal) [80], where animals can become providers of health and activity [81]. As long as animals are present in people’s lives, they offer long-term, additional support for confronting life’s difficulties [82]. Regarding this issue of support, studies have shown that pets are sensitive not only to the emotional signals of human beings but also of their peers [83]. Additionally, they seem to represent points of cohesion within families [66]. Supporting the findings of previous studies that indicate that companion pets are more than just pets, they become family, friends, and resilient factors in people’s lives [49].

Conclusive results were obtained regarding the feeling generated from their interactions with their pet(s) and the benefits they provided to the participants’ well-being from a biopsychosocial perspective, which is in line with previous research [58,84]. Additionally, the applied models showed that living with an animal provided important benefits for the humans, in agreement with the evidence found in recent research [28,85,86,87].

It was observed how coexistence with a pet can awaken different feelings, such as joy [80,88,89,90,91] and love, in their interaction with the subjects, with very high levels seen among those who live with a pet compared to those who do not. As for the feeling of protection, those living with a pet were more likely to feel protected than those who did not live with a pet.

The sociodemographic variable of sex was only shown to be predictive regarding happiness, where a woman had twice as much chance of developing happiness because of interacting with a pet than a man, which is in line with other studies [87,92], where women showed higher scores than men regarding emotional aspects [93]. The other sociodemographic variable par excellence, namely, age, was not found to be significant in any of the logistic regression models.

Marital status was observed to be a predictive variable in several logistic regression models, namely, those concerning love and peace. However, we could not make inferences regarding whether living alone or with others strongly predisposed the participants to having these types of feelings. In the specific case of love, both living as a couple or as a single person showed a predictive level but their values were very similar. Regarding peace, the categories related to marital status, living as a couple, and being separated/divorced were predictive. However, as in the case of feeling love, the values were very similar.

Degree level was a predictive variable for both peace and happiness. In both cases, the higher the educational level (from associate’s degree upward), the greater the possibilities of awakening these feelings through interacting with a pet.

Annual income level was a predictive variable for the feelings of relief, anxiety, and being protected. Regarding relief, people who had an intermediate annual income ($20,000–$50,000) showed a higher level of relief through interacting with their pet(s), although its relevance was low. In the case of anxiety, it was shown that people with medium and low annual incomes (less than $50,001) were more likely to feel anxious in their interaction with their pet(s), which is an aspect that was not evident in those with higher annual incomes. Finally, people with lower annual incomes ($0–$20,000) were more likely to feel protected through interacting with their pet(s) than people with higher annual incomes.

## 5. Conclusions

Throughout the present work, the coexistence with pets in the Puerto Rican society was characterized. The attention given to them was usually shared by other members of the family unit and the food offered to them was, in most cases, specific. Additionally, they spent most of the time at home, just like any other member of the family. Their owners showed affection toward them in different ways.

It is worth noting the high influence the pets had on emotions such as love, joy, and peace. In this sense, the pets were part of human life through very intense relationships and interactions that increased personal welfare in most cases, offering improvements in the human–animal biopsychosocial system.

These elements must be taken into account when considering any measure to be implemented by the Puerto Rican public administrations, as well as other countries, regarding the regulation of the treatment and care of pets, which involves moving away from the idea that pets are not subjects worthy of rights and protection. On the contrary, they are a part of our lives and are regulators of the most elemental feelings and emotions of humans.

In a society that is still cruel to pets in many ways, a change in the ethical, moral, and legal paradigm is urgently needed such that society truly protects and cares for its animals. Administrations must ensure this through legislative changes and by integrating sensitization and awareness into education.

A new line of research that is opening up based on the results obtained in this research field is to investigate the mass euthanasia measures implemented by the government institutions of Puerto Rico because of the increase in the populations of dogs and cats. This cruel situation is still the result of a lack of sensitization and awareness, as well as not perceiving other pets as beings with the same capacity for pain, suffering, anguish, etc., as our species. The problems derived from this fact require research and analysis to be able to face them in a decisive manner based on solid and comprehensive results. This is a holistic question that presents very diverse issues, from the social to the ethical, and that would ultimately have the moral development of our civilization in terms of the way it treats animals as a goal [94,95].

## Figures and Tables

**Table 1:** Demographic details of the participants.

Variable	Answer	Percentage
Gender	Female	79%
	Male	21%
Highest level of education	Up to 11th grade	1%
	High school diploma	21%
	Associate’s degree	14%
	Bachelor’s degree	35%
	Master’s degree	21%
	Doctorate	8%
Type of living situation	House	77%
	Apartment with a garden balcony or terrace	16%
	Apartment without a garden balcony or terrace	6%
	Public housing	0.7%
	Institution (nursing home, shelter)	0.3%
Type of living area	Urban	69%
	Rural	31%
Socioeconomic status (annual income)	$10,000 or less	26%
	$10,001–$20,000	20%
	$20,001–$30,000	20%
	$30,001–$40,000	11%
	$40,001–$50,000	10%
	$50,001–$60,000	4%
	$60,001 or more	9%

**Table 2:** General pet information.

Variable	Answer	Percentage
Any pets in the household	Yes	84%
	No	16%
Source of acquisition	Bought	23%
	Rescued	32%
	Gift	35%
	Adopted	10%

**Table 3:** Companion animal caretaking.

Variable	Answer	Percentage
Who is the primary caretaker of the pet (feeding and water), including visits to the vet?	Myself	22%
	Myself and another member of the family	68%
	Another member of the family	8%
	Employee	0%
	Nobody is clearly defined	2%
What type of food do the pets get?	Leftovers	7%
	I cook them their own food	5%
	Food specific to the species	88%
When you are at home, how much time are you and the pet in the same place?	1 to 4 h	20%
	4 to 12 h	32%
	12 h to the whole day	40%
	Some time every 2 days	2%
	Some time every 3 days	2%
	Sometime during the week	1%
	Sometime during the month	1%
	I do not spend time with them	2%
Where does the pet sleep?	Free inside the house	32%
	In my room	33%
	Free in the garden	23%
	Chained in the garden	6%
	In a crate inside the house	3%
	In a crate outside the house	3%
	Sleeps outside on the streets	0%
	I do not know where it sleeps	0%

**Table 4:** Human–animal companion bond.

Variable	Answer	Percentage
Do you engage in any of these activities with your pet(s)? (Please mark all that apply.)	Play	92%
	Walk	57%
	Stroke	94%
	Talk	89%
	Sports	13%
	Training	22%
	Contests	4%
Identify the principal reason(s) why you have a pet (please mark all that apply).	Security	40%
	Company	91%
	Social status	1%
	Luxury	1%
	Pleasure	42%
	It was a gift	4%
	Money	0.5%
	Work	0.5%
Would you say your pet(s) is(are) part of your family?	Yes	99%
	No	1%

**Table 5:** Summary of the predictive variables of the binary logistic regressions carried out for the significant analyzed feelings.

	β	Sig. (*p*)	Exp (β)	95% CI Exp (β)	
				Lower	Upper
Love					
Lives with a pet	3.003	0.000	20.152	10.443	38.888
Marital status: couple	0.678	0.026	1.969	1.083	3.582
Marital status: single	0.488	0.048	1.630	1.005	2.642
Constant	−0.907	0.010	0.404		
Peace					
Lives with a pet	1.864	0.000	6.450	3.039	13.691
Marital status: couple	0.656	0.000	1.928	1.339	2.775
Civil status: separated/divorced	0.568	0.008	1.765	1.156	2.694
Marital status: single	0.672	0.000	1.959	1.428	2.688
Level of studies: associate’s degree or bachelor’s degree	0.442	0.004	1.555	1.150	2.104
Constant	−2.142	0.000	0.117		
Joy					
Lives with a pet	3.640	0.000	38.094	9.482	153.040
Woman	0.695	0.037	2.004	1.044	3.847
Level of studies: associate’s degree or bachelor’s degree	0.709	0.004	2.032	1.260	3.275
Constant	−1.193	0.012	0.303		
Relief					
Lives with a pet	1.478	0.005	4.385	1.548	12.424
Annual income: $20,001–$50,000	−0.569	0.000	0.566	0.412	0.779
Constant	−1.949	0.000	0.142		
Anxiety					
Annual income: $0–$20,000	2.389	0.021	10.898	1.433	82.875
Annual income: $20,001–$50,000	2.395	0.029	10.964	1.271	94.582
Constant	−5.897	0.000	0.003		
Protection					
Lives with a pet	2.029	0.000	7.604	3.482	16.605
Annual income: $0–$20,000	0.380	0.008	1.462	1.106	1.932
Constant	−1.688	0.000	0.185		
